# Clinical considerations, management and treatment of fever of unknown origin caused by urachal cyst: a case report

**DOI:** 10.1186/1752-1947-8-106

**Published:** 2014-03-25

**Authors:** Vincenzo Bagnara, Salvatore Antoci, Salvatore Bonforte, Giovanna Privitera, Tonia Luca, Sergio Castorina

**Affiliations:** 1 Department of Maternal and Infant Medicine and Radiological Sciences, University of Catania, 95123 Catania, Italy; 2 Fondazione Mediterranea “G.B. Morgagni”, 95125 Catania, Italy; 3 Department of Bio-Medical Sciences, University of Catania, 95125 Catania, Italy

**Keywords:** Incision and drainage, Laparoscopic approach, Regression of urachal cyst, Treatment of infected urachal anomalies, Urachal cyst

## Abstract

**Introduction:**

Urachal cysts are rare congenital anomalies that often prompt referral to the paediatric general surgeon because of their associated complications such as infection, abdominal pain and the young age at presentation. In this report we describe a rare case of fever of unknown origin caused by an urachal cyst which was successfully treated with incision and drainage only. Since the first description of urachal anomalies by Cabriolus in 1550, few cases have been reported and, until now, only one case of infected urachal cyst presenting as fever of unknown origin has been described in the literature. Moreover, the spontaneous resolution of an urachal cyst without excision is extremely rare.

**Case presentation:**

We report our experience in the management and treatment of an infected urachal cyst that occurred in a 12-year-old Caucasian girl who presented to our Department of Paediatric Surgery with a 30-day history of evening fever. The urachal cyst was treated only with incision and drainage through a minimally invasive laparoscopic approach.

**Conclusions:**

The incision and drainage of an infected urachal cyst is a simple and safe procedure. It assures a complete recovery and avoids potential surgical complications related to the total excision of the urachal cyst. This report may provide important clues regarding the management of this rare anomaly and we emphasise the importance for paediatricians, who should consider the possibility that a fever of unknown origin can be caused by an urachal cyst, and for surgeons and urologists, because it suggests that conservative treatment of this rare anomaly should be considered when possible.

## Introduction

The urachus is a fibrous tubular structure that begins narrowing during the fourth and fifth months of gestation. It extends from the anterior bladder wall toward the umbilicus, as the median umbilical ligament, within the space between the peritoneum and transversalis fascia [1]. Urachal anomalies may be present in 2% of the general population and they are classified into four anatomic categories: patent urachus (50%), umbilical-urachal sinus (15%), urachal diverticulum (3 to 5%) and urachal cyst (30%). Usually, urachal anomalies remain asymptomatic unless there are complications such as infection, lithiasis and malignant degeneration. In general, symptoms include fever, lower abdominal pain and palpable suprapubic mass, with or without systemic or laboratory evidence of an infectious process [2]. The diagnosis of these anomalies requires ultrasonography, computed tomography and magnetic resonance imaging. The traditional treatment of an infected urachal cyst is composed of a two-stage approach: an incision and then drainage of the infected cyst followed by secondary excision [3].

In this paper, we describe a case of spontaneous regression of urachal cyst, occurring in a child after incision and drainage only, and we analyse the diagnosis and treatment of this rare disorder. Until now, only one case of infected urachal cyst presenting as fever of unknown origin has been described in the literature [4]. This report is important for paediatricians, who should consider the possibility that a fever of unknown origin can be caused by an urachal cyst, and for surgeons and urologists, in order to consider a conservative treatment of this congenital defect.

## Case presentation

**Figure 1:**
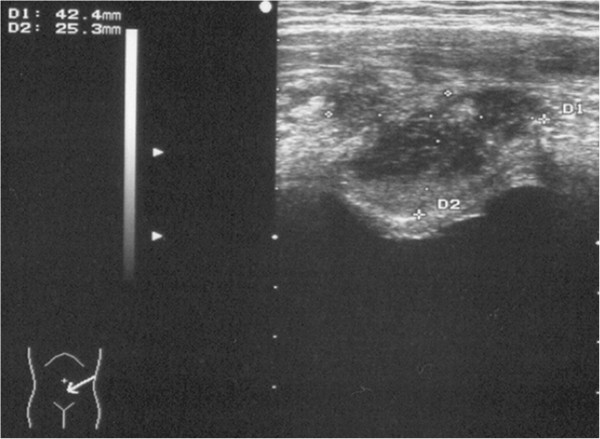
Abdominal ultrasonographic image demonstrating an urachal cystic mass.

A 12-year-old Caucasian girl presented to our Department of Paediatric Surgery with a 30-day history of evening fever (38.5 to 39°C). Her general condition was good. After 7 days of fever, laboratory results showed a white blood cell count of 10,000/mm^3^ and a platelet count of 600,000/mm^3^ of blood. Inflammatory indices were increased: erythrocyte sedimentation rate was 56mm/hour, and C-reactive protein was 208mg/L; microhaematuria was also present (20/high-power field of red blood cells). The results of urinary tests and urine culture were negative. An abdominal ultrasound scan initially revealed no abnormalities and, although a focus of infection was not evident, antibiotics were administered as a preventive measure. After 3 weeks of fever and poor response to antibiotic therapy, prednisone was administered as it was suspected that she had rheumatoid arthritis. After this treatment, she had a remission, but in the following month she complained of suprapubic pain. A physical examination showed painful palpation of her hypogastria and no inflamed lesion on the surface of her skin. A repeat ultrasound evaluation of her abdominal wall revealed abscess formation without any communication with her bladder. In the absence of any umbilicus discharge, we suspected an infected urachal cyst (Figure 1).

**Figure 2:**
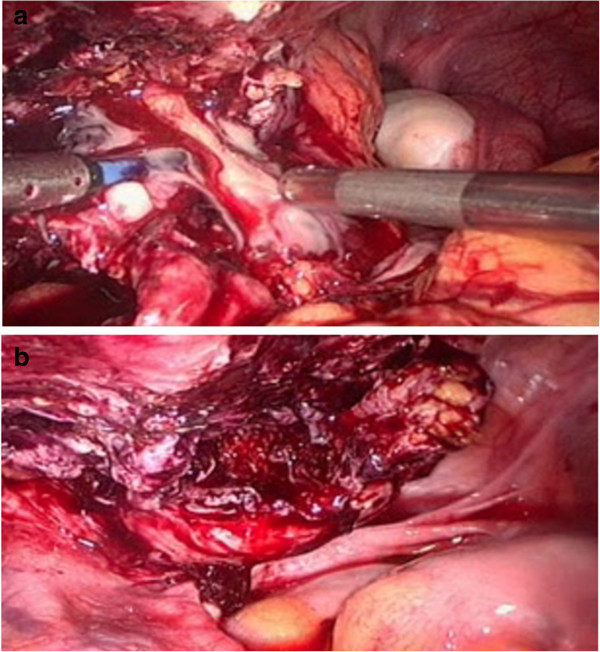
**Intraoperative images of urachal cyst. (a)** Incision and drainage of the cyst. **(b)** Image of the residual cavity after large incision and cleansing.

**Figure 3:**
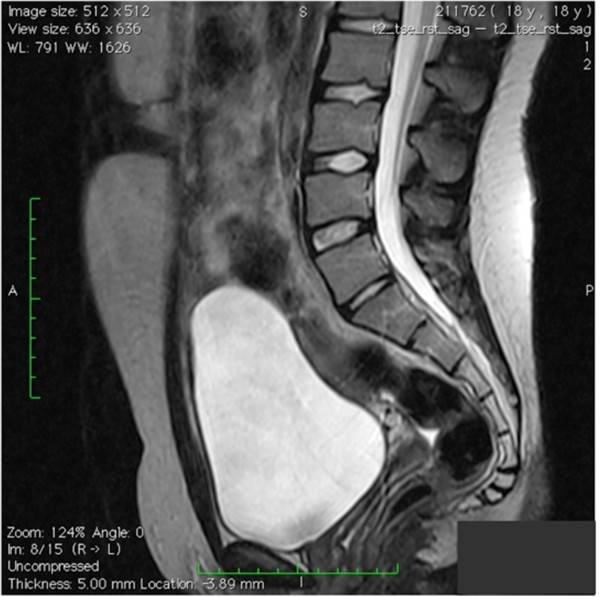
Magnetic resonance imaging scan showing the absence of the urachal cyst 6 years after surgery.

Afterwards, she underwent an immediate exploratory laparoscopy, which confirmed the presence of an infected urachal cyst. Because of inflammation and bleeding, we decided not to excise the cyst but to perform only a large incision and drainage (Figure 2). Tissue specimens were taken for histological examination, which revealed inflammatory infiltrate with no evidence of malignancy and residual mucosa. There were no postoperative complications or recurrences after the incision and drainage of the infected urachal cyst. To our surprise, during the follow-up, echographic studies, performed after 3 and 6 months, showed a gradual reabsorption of the cyst; it was completely reabsorbed after 12 months. Six years later she is well and the urachal cyst has remained absent on magnetic resonance scans (Figure 3).

## Discussion

Urachal cyst is a congenital birth defect characterised by a sinus remaining from the allantois during embryogenesis and is the most common urachal anomaly in the paediatric population. Robert *et al*. noted a physiological regression of urachal anomalies with age. Because urachal remnant diseases are uncommon and manifest with non-specific abdominal or urinary signs and symptoms, surgeons and urologists should be acquainted with the treatment of these rare anomalies, especially in paediatric populations [5]. Knowledge of embryology and anatomy and having a high degree of suspicion are therefore very important to define a correct diagnosis. Although the guidelines reported in the latest edition of Grosfeld’s *Paediatric Surgery* recommend a surgical treatment of infected urachal cysts, spontaneous resolution without the need for surgical excision has been reported [6]. Another surgical option of the management of infected urachal cysts suggests an initial incision and drainage, followed by a later excision of the urachal remnant [7].

In our case we did not perform the second-look surgery. The urachal cyst was completely reabsorbed after the incision and the drainage and, 6 years later, remained absent on subsequent imaging studies. A histological examination demonstrated that there was no mucosa and it did not reveal any sign of malignancy. Even though this histological study regarded only a part of the cyst, we suppose that probably the purulent inflammation destroyed the mucosa lining the cavity of the cyst, thus excluding the possibility of a neoplastic degeneration. Although many surgeons recommend that urachal anomalies are removed to avoid potential malignant transformation later in life, no association has been established between urachal anomalies and urachal carcinoma [8]. Therefore, in our opinion, it seems imprudent to excise urachal remnants in infancy or childhood with the purpose of preventing a risk of developing cancer later in life.

In the literature there is only one study on fever of unknown origin caused by infected urachal cysts [4] and there is very little on treatment with incision and drainage only. In children who have a fever of unknown origin for more than 3 weeks and/or abdominal pain without a specific cause, the possibility of an urachal cyst should therefore be considered and a period of surveillance may be necessary and reasonable before surgery is performed. In our case, for the treatment of an infected urachal cyst, we preferred the laparoscopic approach instead of the percutaneous one. The laparoscopic approach allows a more complete diagnostic balance in which the relation of the cyst with respect to the bladder is better defined, and it allows exploration of the abdominal cavity and its organs. In our opinion, the laparoscopic approach represents a strong treatment of infection. In fact, this approach allows a large incision of the cyst with a complete emptying of purulent material, a better cleansing of the residual cavity and the possibility to take biopsy samples.

Certainly, we do not draw conclusions with a single case, but our experience in surgery, and in particular in laparoscopic techniques [9], has given us convincing reasons to confirm that the performance of the incision and drainage of the infected urachal cyst, through a minimally invasive laparoscopic approach, is a simple and safe procedure. It assures a complete recovery avoiding potential surgical complications related to the total excision of the urachal cyst.

## Conclusions

In conclusion, we report a rare case of fever of unknown origin caused by an infected urachal cyst, which was successfully treated with incision and drainage only. Clinical presentation is non-specific; therefore, a high index of suspicion is required in order to make the diagnosis. This is a rare pathology and, despite the guidelines recommending surgical treatment, we chose a conservative approach. Diagnostic imaging studies performed during the follow-up showed the complete resolution of the cyst. In the light of these data, we emphasise the educational merits of this original case report considering also that very little is present in the literature on this topic.

## Consent

Written informed consent was obtained from the patient, now adult, for publication of this case report and any accompanying images. A copy of the written consent is available for review by the Editor-in-Chief of this journal.

## Competing interests

The authors declare that they have no competing interests.

## Authors’ contributions

VB: design, acquisition of data, data analysis/interpretation, critical revision of the manuscript and approval of the article. SA: acquisition of data, data analysis/interpretation. SB: acquisition of data, data analysis/interpretation. GP: study of literature, drafting of the manuscript. TL: study of literature, drafting of the manuscript. SC: design, acquisition of data, data analysis/interpretation, critical revision of the manuscript and approval of the article. All authors read and approved the final manuscript.
